# Tunable Technologies for the Glioma Tumor Microenvironment: A Comprehensive Review on Bench-to-Bedside Neurosurgical Advances

**DOI:** 10.3390/brainsci16060578

**Published:** 2026-05-29

**Authors:** Eshita Sharma, Julieta Serobyan, Numa Rajab, Aisha Rizwan Ahmed, Santosh Guru, Michael K. Lim

**Affiliations:** 1 David Geffen School of Medicine, University of California Los Angeles, Los Angeles, CA 90095, USA; egsharma@mednet.ucla.edu (E.S.); jserobyan@mednet.ucla.edu (J.S.); 2 Department of Medicine, Sulaiman Al Rajhi University, Al Bukayriyah 52726, Saudi Arabia; nuparveenr@gmail.com; 3 Department of Medicine, Jinnah Medical and Dental College, Karachi 74800, Pakistan; aisha.riz.ahmed@gmail.com; 4 School of Clinical Medicine, University of Cambridge, Cambridge CB2 1TN, UK; sg928@cam.ac.uk; 5 Department of Neurosurgery, Stanford University School of Medicine, Stanford, CA 94305, USA

**Keywords:** glioblastoma, tumor microenvironment, fluorescence-guided surgery, Raman spectroscopy, focused ultrasound, convection-enhanced delivery, nanotechnology, blood–brain barrier

## Abstract

**Highlights:**

**What are the main findings?**

- The paradigm of glioma surgery is transitioning from a conventional debulking approach to a “biology-integrated” model that emphasizes the identification and targeting of specific metabolic and molecular niches within the tumor microenvironment.
- Surgeons can now use cutting-edge tools like Raman spectroscopy for real-time molecular fingerprinting and focused ultrasound for modulating the blood–brain barrier to treat complex tumor niches that were previously unreachable with standard methods.

**What are the implications of the main findings?**

- Tailoring surgical methods to the tumor’s biological characteristics increases the precision of surgical resections. This way, we can protect the healthy brain tissue while also targeting the tumor regions of most danger.
- For clinical translation to be successful, trial designs will need to focus more on how drugs are distributed in space and how they interact with biology than on traditional volumetric metrics. This will require better integration of different types of technology in the operating room.

**Abstract:**

Gliomas remain among the most treatment-resistant malignancies of the central nervous system. Glioblastoma (GBM), the most aggressive adult-type diffuse glioma, is associated with persistently poor survival despite maximal safe resection followed by chemoradiation. Gliomas do not grow in isolation. Work over the past twenty years has dismantled the older tumor-centric view of glioma biology, replacing it with a model in which malignant cells operate within a tumor microenvironment (TME) composed of immune, vascular, stromal, and neural elements that together govern disease behavior. What makes the glioma TME so difficult to treat is not just its composition of immune cells, vasculature, stroma, and neurons, but the fact that these elements are arranged unevenly across the tumor. Different regions harbor different cellular mixtures and signaling environments, and, as a result, different vulnerabilities to therapy. Cytoreduction has not lost its importance, far from it. However, the same surgical window now also serves a different purpose; it lets the surgeon see which tissue is biologically dangerous rather than just visually abnormal, locate the true edge of infiltration, and get therapeutics past a blood–brain barrier (BBB) that has historically locked them out of the brain. This review examines two technology domains, including: (1) optical theranostics (5-aminolevulinic acid fluorescence-guided surgery, fluorescein-guided visualization, Raman spectroscopy, and stimulated Raman histology); and (2) blood–brain barrier disrupting technologies. The direction they collectively point toward is a version of glioma surgery that is guided less by anatomy and more by the biology of the tumor itself.

## 1. Introduction

The most common primary malignant tumors of the central nervous system, gliomas, are also among the hardest to treat. Glioblastoma (GBM) is one of the most therapeutically resistant cancers in medicine. GBM patients survive a median of 14–16 months, even after maximal safe resection, radiotherapy, and temozolomide [1,2,3,4]. Better surgical technique, better radiation, better drugs: none of it has changed that number much. For most of that history, glioma biology was read through a tumor-centric lens—malignant glial cells were the dominant drivers. Everything else was secondary. That view has been dismantled over the past twenty years. Gliomas, in fact, are not just collections of malignant cells. They are ecosystems. Stromal elements, vasculature, immune populations, and neural components are all embedded in the tumor, and all contribute to how it grows, invades, resists treatment, and comes back [5,6,7,8].

The cellular composition of the glioma tumor microenvironment (TME) is broad. Tumor-associated macrophages and microglia (TAMs), endothelial cells, pericytes, astrocytes, neurons, oligodendrocyte precursor-like elements, fibroblast-like stromal populations, and extracellular matrix constituents have all been identified within it [5,6,7,8,9,10]. None of these are bystanders. They form networks that sustain the tumor, and they do so differently depending on where in the tumor they sit. TAMs alone can account for 30–50% of the total cellular mass in GBM. They arise from two sources: resident microglia already present in the brain and bone marrow-derived macrophages pulled into the tumor bed from the periphery [9,10,11,12,13]. Single-cell and spatial transcriptomic data have complicated the picture further. TAMs near blood vessels do not resemble TAMs in hypoxic or necrotic zones. The phenotype changes again at the infiltrative edge, and it changes again between a newly diagnosed tumor and a recurrence [9,11,14]. There is no single immunologic state in a glioma; there are many, and they map onto specific spatial and microenvironmental regions of the tumor.

Within this immunosuppressive landscape, TAMs secrete cytokines and growth factors, including transforming growth factor-β (TGF-β), interleukin-10 (IL-10), and colony-stimulating factor-1 (CSF-1), that suppress cytotoxic T-cell function and promote angiogenesis and mesenchymal transition [14,15,16,17,18]. Innate immune checkpoint signaling has also arisen as a central mechanism of phagocytic escape. Tumor-cell expression of CD47 inhibits macrophage and microglial engulfment through interaction with signal regulatory protein-α (SIRPα), while the CD24–Siglec-10 axis constitutes an additional “don’t-eat-me” pathway playing a role in immune evasion in glioma and other solid tumors [19,20,21,22]. These discoveries carry direct technological implications: they suggest that future neurosurgical and perioperative platforms may need to target immune architecture and myeloid reprogramming rather than tumor bulk alone.

The vascular compartment represents another defining feature of the glioma microenvironment. Gliomas exhibit highly abnormal vascular architecture characterized by tortuous vessels, heterogeneous perfusion, microvascular proliferation, and increased permeability driven in part by vascular endothelial growth factor (VEGF)-dependent signaling [23,24,25,26]. Gliomas build blood supply through several routes beyond classical sprouting angiogenesis. They co-opt host vessels that are already there. They generate entirely new vasculature through vasculogenesis. They form mimicry channels that look like vessels but are not, and glioma stem-like cells can themselves take on endothelial-like properties [24,27,28]. What this produces is a vascular network that is structurally and functionally abnormal, and that abnormality has consequences for how drugs reach the tumor and how the tumor itself behaves. Although enhancing regions of GBM commonly exhibit blood–brain barrier (BBB) disruption, infiltrative non-enhancing margins may preserve a relatively intact BBB or blood–brain–tumor barrier, thereby shielding residual invasive cells from systemic therapies [26,29,30].

**Figure 1:**
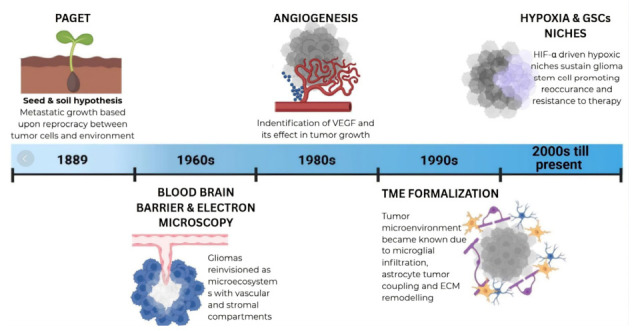
Timeline of key discoveries shaping glioma microenvironment biology. VEGF—vascular endothelial growth factor; TME—tumor microenvironment; HIF1-α—hypoxia-inducible factor-1 alpha; ECM—extracellular matrix.

Hypoxia is just as important. Gliomas frequently outgrow their own blood supply, and the oxygen-starved tissue that results activates hypoxia-inducible factor (HIF)-dependent transcriptional programs: HIF-1α– and HIF-2α–mediated angiogenesis, glycolytic reprogramming, resistance to apoptosis, and increased invasiveness [31,32,33,34,35]. These same hypoxic and peri-necrotic zones tend to be where glioma stem-like cells concentrate, and the phenotypic plasticity that hypoxia promotes is one reason tumors come back after treatment [33,34,35,36]. Multi-omic and spatial transcriptomic work has added another layer to this picture. Glioma cell states, immune suppression, and vascular signaling are not scattered at random. They sort into recognizable habitats—perivascular, peri-necrotic, inflammatory, and neuron-associated—and a therapy that works in one may fail in another [37,38,39,40,41]. That is not a minor detail for surgeons. If the tumor is an ecosystem with distinct neighborhoods, then operating on it means intervening in those neighborhoods, not removing a uniform mass (Figure 1).

This has consequences for how we think about surgery. The traditional rationale for operating on a glioma was cytoreduction: take out as much tumor as safely possible without damaging the eloquent brain [42,43,44,45,46]. That rationale has not been abandoned, but it is no longer sufficient on its own. The question that matters biologically is not just how much tumor was resected, but which parts of the tumor were resected. A surgeon can achieve gross total removal of every contrast-enhancing voxel and still leave behind the compartments most responsible for recurrence—infiltrative margins, hypoxic zones, stem-cell niches, macrophage-dense regions, and tissue sitting behind an intact blood–brain barrier [29,33,42,43,44,45,46].

Biology-integrated neurosurgery has grown out of this problem. Intraoperative imaging and delivery technologies have reached a point where the surgeon can see metabolic gradients in the tissue, locate infiltrative boundaries that are invisible under white light, characterize tissue composition on the spot, and alter vascular permeability to change how drugs distribute through the tumor bed [47,48,49,50,51]. Among the broad set of emerging neuro-oncologic tools, two technology domains have demonstrated particularly strong clinical translation: optical theranostic technologies, which reveal metabolic, vascular, and molecular features of tumor tissue, and BBB disruption and targeted deployment platforms, which seek to expose otherwise pharmacologically protected niches to therapy [47,48,49,50,51,52,53,54,55,56,57,58].

This review evaluates the mechanistic basis and translational findings supporting these technology domains and discusses how they enable neurosurgeons to interrogate and modulate the glioma TME. The picture that emerges from all of this is a version of glioma surgery that has outgrown its original purpose. The operation is still about removing the tumor. But it is also, now, about seeing what kind of tissue is there, sampling it, disrupting the microenvironment around it, and using the surgical window to deliver therapy that would not otherwise reach the disease.

## 2. Optical Theranostics

### 2.1. Biological Basis for Optical Visualization of the Tumor Microenvironment

Optical imaging is where this shift is most visible in practice. Standard white-light microscopy shows the anatomy: what looks like a tumor versus what looks like brain. It does not show metabolism, vascular permeability, or molecular composition, and in glioma, those are often what matter most [47,48,49,50,51,59,60,61]. The operative problem in glioma is not a boundary. It is a gradient of infiltration density that tapers, a reactive parenchyma intermixed with tumor cells, and a barrier function that shifts from one millimeter to the next. None of that is visible under white light. Optical technologies make some of it visible, and in doing so, give the surgeon something to act on intraoperatively.

The most established mechanism underlying optical visualization in glioma is altered mitochondrial heme biosynthesis following administration of 5-aminolevulinic acid (5-ALA). Glioma cells have dysregulated porphyrin pathway activity, and after oral administration of 5-ALA, they preferentially accumulate protoporphyrin IX (PpIX), a fluorescent metabolite that glows red under blue-violet excitation [47,48,49,50,51]. The accumulation is not accidental. Reduced ferrochelatase activity slows the normal conversion of PpIX to heme, and broader derangements in mitochondrial metabolism and bioenergetics compound the effect [49,50,51,52]. PpIX fluorescence, then, is not a stain. It is a metabolic signal. It reports on the bioenergetic state of the tissue.

The signal is not uniform, and that turns out to be diagnostically useful. Histopathologic correlation studies have found that the brightest regions of PpIX fluorescence tend to sit on top of the most densely cellular, most proliferative tissue, which is not surprising, given that those cells accumulate the most PpIX [49,52,53]. What is more useful to the surgeon is what happens at the edges, where fluorescence dims. That tissue is usually infiltrative, with fewer tumor cells per unit volume, and it is precisely the tissue that is easiest to miss or misjudge under white light. So the fluorescence gradient, imperfect as it is, gives the surgeon a crude spatial readout of how the microenvironment is organized across the resection field. The bright core is the densely cellular, metabolically active compartment that shows up on contrast-enhanced MRI. The dim periphery marks the transition zone: the tumor cells and brain interdigitate, density falls off, and biology changes [49,53].

Optical visualization also captures the vascular dimension of the TME. High-grade gliomas frequently exhibit BBB breakdown and abnormal vascular permeability, enabling fluorophores, such as fluorescein sodium, to accumulate in enhancing tumor tissue [54,62,63]. Unlike 5-ALA, fluorescein is not a tumor-selective metabolic agent, but a permeability tracer, which makes it informative about the angiogenic and barrier-disrupted compartments of the microenvironment rather than about malignant-cell biology per se [54,62].

There is also a class of optical methods that does not require any exogenous agent at all. Raman spectroscopy and stimulated Raman histology (SRH) work by measuring vibrational signatures of lipids, proteins, and nucleic acids in fresh, unprocessed tissue [55,56,57,58,64,65]. The biochemical fingerprint that comes back is specific enough to separate tumor from normal brain, and recent iterations of these platforms can do it fast enough to be useful during surgery. Some can now infer molecular subtype as well [56,57,58,64]. Between fluorescence-based methods and label-free spectroscopy, the optical tools available to the surgeon now cover three distinct dimensions of tumor biology: how the tissue is metabolizing, where the barrier is leaking, and what the tissue is made of at a molecular level. This sentence clearly demonstrates how tumor biology translates into intraoperative decision making. The following subsections will examine each modality in detail.

### 2.2. 5-Aminolevulinic Acid Fluorescence-Guided Surgery

No optical technology in glioma surgery has been studied more than 5-ALA. The mechanism is straightforward: after oral administration, 5-ALA is metabolized through the heme biosynthesis pathway into PpIX, which builds up preferentially in malignant glioma cells because their mitochondrial metabolism is abnormal and their ferrochelatase activity is low [47,48,49,50,51,52,53]. That preferential accumulation is what makes the fluorescence tumor-selective, and it is what justified bringing the technique into the operating room.

The trial that changed the field was the Stummer Phase III study. It was a randomized, multicenter comparison of 5-ALA-guided resection against conventional white-light surgery in malignant glioma. Surgeons using 5-ALA achieved complete resection of contrast-enhancing tumor 65% of the time, compared with 36% under white light, and their patients had better six-month progression-free survival [48]. Those resection rates mattered, obviously. But what made the Stummer trial a turning point was less about percentages and more about what they implied: that biology, not just anatomy, could guide the surgeon’s hand in real time, and that patients were better off for it. Pooled analyses and systematic reviews published afterward confirmed the pattern that 5-ALA was consistently associated with more complete resections and longer progression-free survival, with some data suggesting an overall survival benefit as well [66,67,68,69].

Contemporary data have further validated these results under modern surgical conditions. In the RESECT study, a French multicenter randomized phase III trial, Picart and colleagues demonstrated that 5-ALA significantly increased gross total resection rates in comparison to white-light microsurgery (79.1% vs. 47.8%) without increased neurological morbidity [70]. Schupper et al. then validated these findings in a prospective multicenter U.S. study carried out after FDA approval. Visible PpIX fluorescence proved sensitive and had a strong positive predictive value for high-grade glioma across participating centers following FDA approval [71]. Notably, these trials demonstrate that the benefit of 5-ALA persists even when layered onto contemporary neuronavigation workflows, an important finding given that earlier critics had questioned whether modern imaging would render fluorescence guidance redundant.

The significance of 5-ALA goes beyond volumetric resection gains when considered through the lens of tumor microenvironment biology. Quantitative fluorescence data bear this out; the brightest tissue is consistently the most densely cellular, the most proliferative, and the most metabolically deranged [49,52,53]. 5-ALA, in other words, is not just helping the surgeon see the tumor. It is reporting on which parts of the tumor are biologically the most aggressive. Visible fluorescence generally marks the biologically active core compartment, while fainter fluorescence at resection margins reflects the gradual transition into infiltrative, lower-density disease where tumor and brain interdigitate [49,53].

**Figure 2:**
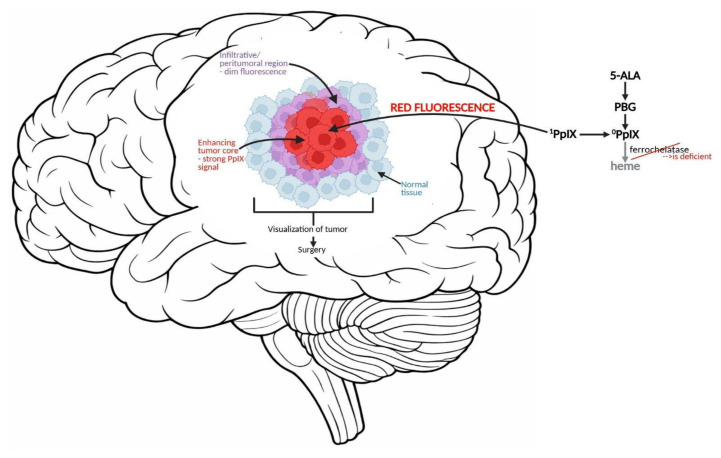
5-ALA/PpIX-induced fluorescence of tumor tissue and key zones of demarcation. 5-ALA—5-aminolevulinic acid; PBG—porphobilinogen; PpIX—protoporphyrin IX. PpIX superscript values indicate relative fluorescence intensity, where 1 represents high fluorescence and 0 represents absent or minimal fluorescence.

5-ALA is useful outside of open resection, too. In stereotactic biopsy, fluorescence helps the surgeon land the needle in the most diagnostically informative tissue, which matters especially in tumors, where histology varies from one region to the next and where missing a focus of high-grade transformation can change the entire treatment plan [50,53]. There is a technical limitation here, though: the surgeon can only act on fluorescence that is actually visible. PpIX does accumulate in infiltrative, lower-density tissue at the tumor margin, but at concentrations the eye cannot reliably detect. Spectroscopic instruments can [49]. That gap between what the eye sees and what the spectrometer measures is, in a sense, the next problem to solve. If quantitative PpIX detection matures into a reliable intraoperative tool, the result would be something closer to biologic mapping of the tumor–brain interface than anything the operating room currently offers (Figure 2).

5-ALA is not without problems. It works best in high-grade, contrast-enhancing gliomas. In low-grade diffuse disease and non-enhancing margins, the signal is weaker and less dependable [47,49]. Prior treatment, tissue depth, the optics of the surgical microscope, and photobleaching all affect what the surgeon sees. None of that changes the fact that 5-ALA is, currently, the clearest example of a laboratory finding about tumor metabolism being turned into something a neurosurgeon can use at the bedside to make better decisions [47,48,66,67,68,69,70,71].

### 2.3. Fluorescein-Guided Surgery

Fluorescein sodium has followed a distinctly different translational trajectory than 5-ALA and should be perceived through a different biologic lens. Whereas 5-ALA reports on altered intracellular metabolism, fluorescein accumulates primarily inside regions of BBB disruption and increased vascular permeability [54,62,63]. In glioma, this makes fluorescein most informative about the vascular and barrier-dysregulated compartment of the TME, a biologic axis largely orthogonal to the metabolic information provided by PpIX fluorescence.

With dedicated microscope filters now widely available, fluorescein-guided surgery has become a practical and comparatively inexpensive adjunct for high-grade glioma resection. Acerbi and colleagues, in the multicenter prospective phase II FLUOGLIO study, demonstrated high rates of complete tumor removal with acceptable sensitivity and specificity for tumor detection and no major fluorescein-related adverse events [62]. Neira et al. went a step further, reporting that fluorescein was helpful even at infiltrative margins, including tissue that did not enhance on preoperative imaging but that turned out, on pathology, to contain tumor [63]. If that holds up in larger studies, fluorescein’s role may not be limited to the enhancing core. Katsevman et al. reported similar findings, with improved resection targets and longer survival observed in observational data from fluorescein-assisted glioblastoma surgery [72].

What limits fluorescein biologically is the same thing that makes it interesting conceptually. It does not label tumor cells the way 5-ALA does. It labels leaky vasculature. That means it is good at finding the angiogenic, barrier-disrupted parts of the tumor—the regions where vessels are abnormal, and permeability is high—but it is probably not the right tool for the infiltrative margin, where the barrier may still be relatively intact, and the problem is scattered tumor cells rather than vascular breakdown [54,62,63]. The two agents are not in competition with each other. They are reporting on different things. And there is an obvious implication: using both at once, one tracking metabolism and the other tracking vascular permeability, would give the surgeon a more layered picture of the microenvironment than either one provides on its own. A summary of the key findings regarding these two agents is presented in the table below (Table 1).

### 2.4. Raman Spectroscopy and Label-Free Optical Imaging

Raman spectroscopy works by a completely different principle. There is no fluorophore involved, no drug the patient takes beforehand, no molecule that needs to accumulate selectively in the tumor. Instead, the technique measures vibrational spectra generated by molecular bonds in the tissue itself, and those spectra differ enough between tumor and normal brain to allow discrimination based on intrinsic biochemistry alone [55,64]. This capacity makes Raman especially suitable for interrogating the tumor–brain interface, where infiltrative disease may evade conventional visual inspection and where fluorophore-based signals are often weak or absent.

Jermyn and colleagues provided early translational evidence that Raman spectroscopy could detect brain cancer in fresh tissue with high diagnostic accuracy [55]. Stimulated Raman histology (SRH) subsequently represented a substantial advance by translating raw spectroscopic signals into a rapid, workflow-compatible intraoperative pathology platform. Hollon et al. demonstrated the clinical potential of this approach in a multicenter study: their machine-learning-assisted SRH platform classified tissue about as accurately as conventional histopathology, but it did so in minutes. This represented a significant improvement over the turnaround time for frozen sections [56]. Subsequent work has expanded this paradigm in important directions. In a direct comparison with 5-ALA-guided surgery, Livermore et al. presented superior Raman detection of infiltrative tumors in fresh tissue samples, particularly at resection margins [64]. Reinecke et al. then showed that SRH could support not only intraoperative classification of tumorous versus nontumorous tissue but also clinically relevant molecular subtyping in stereotactic biopsy specimens [57].

What Raman-based methods are doing, in effect, is changing the question the surgeon can ask during the operation. Older intraoperative tools asked a binary question: tumor or not tumor? SRH and related platforms are starting to answer a different one—what kind of tissue is this, and what molecular program is driving it [56,57,64]? That distinction matters more than it might seem. Spatial transcriptomic studies have now shown that glioblastomas are not molecularly uniform masses but collections of biologically distinct niches, each with its own therapeutic profile [37,38,39,40,41]. The problem has always been that those findings come from the laboratory, after the operation is over. If Raman-based imaging can approximate that level of tissue characterization while the patient is still on the table, it becomes the thing that connects molecular biology to surgical decision making. No other optical platform currently in development is as close to doing that.

## 3. Blood–Brain Barrier Disruption and Targeted Delivery Technologies

### 3.1. The Blood–Brain Barrier as a Therapeutic Barrier

If optical theranostics tackles the problem of seeing biologically relevant tumor tissue, the technologies in this section address a different problem: delivering effective therapy to it.

The blood–brain barrier stands at the center of this problem. Endothelial tight junctions, pericytes, astrocytic endfeet, and basement membrane components form a neurovascular interface that restricts both paracellular and transcellular passage of therapeutic agents into the central nervous system [26,29,73,74]. Contrast enhancement on MRI tells the surgeon that the barrier is disrupted somewhere, but not that it is disrupted everywhere. The reality is patchwork. Some parts of the tumor, usually the core, have highly permeable vasculature. Other parts, particularly the infiltrative margins, retain a BBB, or blood–brain–tumor barrier, that is largely intact [12,26,29,30,73]. Those two conditions can exist centimeters apart in the same patient.

This has real consequences for treatment. Where the barrier holds, cytotoxic drugs, antibodies, and large biologics do not get in or do not get in at adequate concentrations. The infiltrative cells sitting behind that intact barrier are pharmacologically shielded—they survive not because they are resistant to the drug but because the drug never reaches them [12,26,29,73]. Meanwhile, the leaky core vasculature creates its own set of problems: edema, hypoxia, high interstitial pressure, and erratic nutrient supply. Each of those conditions generates a different local microenvironment with different biology and different treatment susceptibility [23,26,74]. The BBB is not just a wall that keeps drugs out. It is a primary reason for the tumor microenvironment’s organization and a major factor in why the invasive edge of a glioma consistently represents the site of treatment failure.

That problem, differential drug exposure dictated by barrier geography, is what the technologies in this section are trying to solve. They go about it in different ways. Some open the BBB transiently. Some bypass it altogether by delivering drugs directly into the brain. Others try to engineer carriers that can cross it on their own [29,30,73,74,75,76,77]. The goal, though, is the same in every case: get the therapeutic agent into the tumor compartments that systemic delivery currently cannot reach. The subsections that follow examine three such strategies, focused ultrasound-mediated BBB disruption, convection-enhanced delivery, and nanoparticle-based drug delivery, each of which attacks the BBB problem from a different direction.

### 3.2. Focused Ultrasound-Mediated BBB Disruption

**Figure 3:**
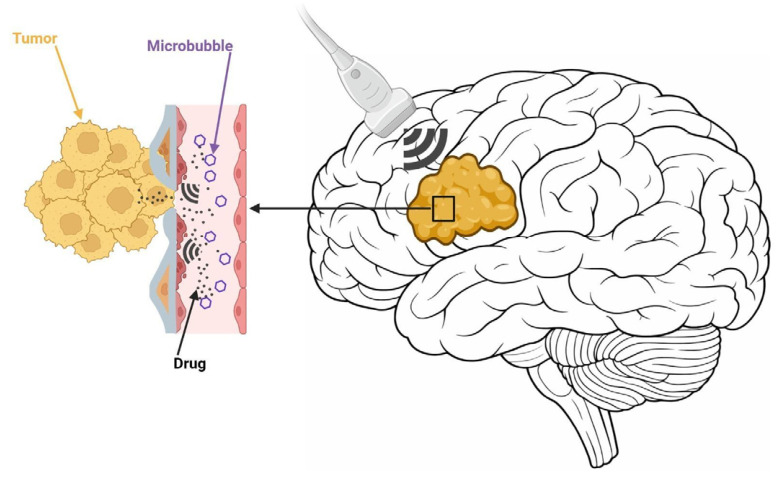
Mechanism of action of focused ultrasound on brain tumors.

Of the three, focused ultrasound has the longest clinical track record (Figure 3). The technique pairs low-intensity pulsed ultrasound with intravenous microbubbles to open the BBB locally and reversibly [75,76,77,78,79]. The mechanism is mechanical. Ultrasound causes the microbubbles to oscillate, and that oscillation stresses the endothelium. Tight junctions widen transiently, vesicular transport increases, and for a window of time, the barrier lets through molecules that it normally would not [75,76]. In animal models, this has translated into higher intratumoral concentrations of chemotherapeutics, antibodies, and nanoparticle-based agents. But there are also hints of something beyond simple drug delivery. Focused ultrasound (FUS) appears to change immune-cell trafficking and vascular signaling in the sonicated region, which raises a question the field has not fully answered yet: whether opening the barrier is itself a biologic intervention, not just a pharmacokinetic one [75,76,77].

Clinical translation has accelerated over the past several years. Mainprize et al. first established the safety and feasibility of noninvasive MRI-guided focused ultrasound for opening the BBB in patients with brain tumors [78]. Anastasiadis et al. pushed the technique into more clinically relevant territory. Working with infiltrating glioma patients, they used MRI-guided, acoustic-emission-controlled FUS to open the BBB specifically in non-enhancing tumor regions, the infiltrative tissue where the barrier is normally still intact. Local fluorescein accumulation increased in the sonicated areas, which was the first direct confirmation that FUS could improve drug delivery into the margins most responsible for recurrence [79]. This was a particularly important advancement because it moved the field beyond augmenting core disease, where permeability is already partially compromised, and into the infiltrative margin most relevant to recurrence.

With implantable ultrasound systems, repeated outpatient BBB opening became feasible, eliminating the need for transcranial procedures at each session. In the first-in-human SonoCloud study, Idbaih, Canney, Belin, and colleagues established the feasibility and safety of repeated monthly skull-implantable pulsed ultrasound treatments in recurrent GBM [73]. Sonabend and colleagues subsequently showed that a nine-emitter implantable device could safely enhance delivery of albumin-bound paclitaxel, with pharmacokinetic analyses demonstrating substantially higher drug concentrations in sonicated peritumoral brain [76]. Larger phase I/II datasets have since confirmed that repeated multi-emitter BBB opening is feasible when combined with carboplatin and other systemic agents [80]. The strongest signal to date comes from the BT008NA trial. Woodworth and colleagues combined microbubble-enhanced transcranial FUS with temozolomide in high-grade glioma patients, confirmed BBB opening at every treatment session, and reported survival outcomes that compared favorably against matched controls [81]. It was the first time an FUS trial had produced a comparative survival result.

The cumulative picture is that FUS is becoming more than a way to get drugs across the barrier. Barrier disruption changes vascular transport. It may change immune-cell trafficking. It almost certainly changes the local microenvironment in ways that are not fully cataloged yet [75,76,77,79,81]. Whether those changes are therapeutically useful on their own—independent of whatever drug is being delivered—is an open question. So are the technical details: what sonication parameters are optimal, how uniform and how large the opening needs to be, how different drugs interact with the opened barrier, how long the effect lasts, and whether the immune and stromal changes that FUS produces translate into survival benefits that hold up over time [75,76,81]. FUS will not become a standard part of neurosurgical practice until those questions have answers.

### 3.3. Convection-Enhanced Delivery

Convection-enhanced delivery (CED) takes the opposite approach to FUS. Instead of opening the BBB, it goes around it. A stereotactically placed catheter delivers therapeutic agents directly into the brain parenchyma under positive pressure, and the resulting bulk flow distributes the drug through the interstitial space over distances that passive diffusion could never cover [82,83,84,85,86]. The match between CED and glioma biology is intuitive: the compartments that matter most for recurrence, infiltrative margins, the tissue surrounding the resection cavity, and deep niches harboring residual cells, are exactly the compartments that systemic therapy has the hardest time reaching [84,85,86].

The conceptual foundation was established in 1994 by Bobo and colleagues, who demonstrated that bulk-flow infusion could distribute macromolecules through brain tissue over clinically meaningful distances [82]. The engineering turned out to be harder than the concept. Chen, Lonser, Morrison, Oldfield, and others showed that distribution volume is sensitive to almost everything, including infusion rate, catheter diameter, whether the infusate refluxes back along the catheter track, how well the tissue seals around the tip, and the geometry of the target itself [83]. The lesson was that in CED, how you deliver the drug is inseparable from whether it works. Local therapy fails not only when the drug is ineffective but also when the relevant microenvironmental compartment is inadequately covered.

The most prominent clinical test of this principle was the PRECISE phase III trial, in which Kunwar and colleagues compared CED of cintredekin besudotox (IL13-PE38QQR) with Gliadel wafers in recurrent glioblastoma [84]. CED did not win. There was no overall survival advantage. But the post hoc story was more interesting than the primary endpoint. Sampson et al. went back and looked at where the catheters had actually been placed and how much peritumoral tissue they had actually covered, and the answer, in many cases, was not enough [85]. Catheter positioning varied widely between patients and was frequently suboptimal. This finding carries a larger lesson for glioma therapeutics: in spatially heterogeneous tumors, a biologically rational agent can appear ineffective if it never reaches the relevant tumor habitat. Drug mechanism and delivery geometry are inseparable.

The technical problems with CED have not gone away, but they are being addressed. Spinazzi et al. built the first system designed for chronic, continuous delivery; they implanted pumps that infused topotecan into recurrent glioblastoma over several weeks rather than in a single operative session. It worked. Patients tolerated it, and the drug produced visible effects in the target tissue [87]. Other groups have taken the platform in different directions, including real-time MRI guidance to track where the infusate is actually going, radiotherapeutic payloads, and oncolytic viruses [87,88,89]. CED is no longer the fragile, inconsistent technique it was a decade ago, though it is not yet routine either.

What makes CED appealing from a microenvironment standpoint is that the surgeon controls where the drug goes. Systemic chemotherapy cannot do that. A catheter placed in the peritumoral margin can deliver cytotoxic agents, anti-angiogenic compounds, immunomodulatory drugs, or agents that target glioma stem-cell biology directly into the tissue harboring residual disease [84,85,86,87,88,89,90]. Surgically informed catheter placement within peri-cavitary tissue may enable spatially directed modulation of the postoperative glioma microenvironment. The payload is almost secondary to the principle. The hard part, and it remains hard, is matching the catheter position and infusion volume to the actual geography of whatever tumor biology is left behind after resection.

### 3.4. Nanoparticle-Based Drug Delivery

The idea behind nanoparticles is different from either FUS or CED. The barrier stays closed. Nothing is injected directly into the brain. Instead, the particle itself is designed to get across [74,91,92,93,94,95]. The range of platforms under investigation is wide—polymeric nanoparticles, liposomes, micelles, dendrimers, inorganic particles, biomimetic, and hybrid constructs—and they are being engineered to solve several problems at once: surviving systemic circulation long enough to reach the brain, protecting the therapeutic payload from early metabolism, crossing the BBB, and then accumulating preferentially in tumors rather than in healthy tissue [74,91,92,93,94,95]. However, unlike simpler drug-delivery vehicles, nanoparticles can function as programmable platforms. We can tune these carriers’ behavior to the biologic conditions of the TME itself.

Several features of glioma biology make this tunability relevant. Abnormal tumor vasculature and intermittent permeability may permit some degree of passive nanoparticle accumulation. However, the enhanced permeability and retention (EPR) effect is considerably less reliable in brain tumors than in many extracranial cancers, precisely because BBB integrity varies so markedly across the tumor landscape [12,26,29,74]. Surface functionalization with ligands targeting endothelial receptors, tumor-cell antigens, or macrophage-associated markers is able to enhance active transcytosis, selective retention, or delivery targeted to immune cells [74,91,92,93,94,95]. What may matter most for the tumor microenvironment, though, is that nanoparticles do not have to release their payload on a fixed schedule. Release can be made conditional, triggered by acidic pH, local enzymatic activity, oxidative stress, or an external stimulus like ultrasound [74,91,92,93,94]. A particle that releases the drug only when it encounters the biochemical conditions of a hypoxic niche or an immunosuppressive zone is not just a carrier. It is a platform whose behavior is coupled to the biology of the tissue it sits in.

That is what sets nanoparticles apart. A particle can be built so that it preferentially finds the proliferative core, or the perivascular niche, or the macrophage-dense zones where immune suppression is most entrenched, depending on how its surface chemistry and payload are configured [91,92,93,94,95]. Newer lipid-based and PLGA-based systems, for example, are being developed specifically to cross the BBB, target glioma stem cells, and counteract the immunosuppressive TME [92,93,94,95]. In orthotopic GBM models, other preclinical platforms, including synthetic protein nanoparticles and ligand-decorated liposomal systems, have demonstrated encouraging results [91,96,97]. The field is clearly moving away from trial-and-error formulation. Particle design is increasingly dictated by what is known about the microenvironment the particle is meant to enter, which receptors to target, which conditions to exploit for drug release, and which compartment to prioritize.

Getting these systems to work in patients has been a different story. The BBB is not crossed consistently. The reticuloendothelial system clears particles before they reach the brain. Manufacturing at clinical grade is difficult. Off-target accumulation remains a problem. And confirming that a nanoparticle actually reached the tumor in a living human brain, rather than in a mouse flank model, is still extraordinarily hard to do [74,91,92,93,94,95]. Nanoparticle-based therapy is further from the bedside than either 5-ALA or focused ultrasound. They represent one of the most active translational frontiers in glioma therapeutics, but the gap between elegant preclinical design and reliable clinical performance has yet to be closed. Nanoparticle platforms may ultimately support biologically informed surgery through simultaneous intraoperative visualization, molecular targeting, and localized therapeutic delivery within infiltrative tumor margins.

With these three delivery strategies surveyed alongside the optical technologies examined earlier, we now turn to the cross-cutting principles that emerge when these domains are considered together (Table 2).

## 4. Discussion

The technologies reviewed in this article share a common trajectory: each originated from an insight into glioma microenvironment biology and was subsequently engineered into a tool that allows surgeons to act on that insight intraoperatively or perioperatively. What ties them together is a change in what glioma surgery is for. Removing tumor volume has not become less important. But the field is moving toward a version of surgery where the operation is also a chance to read the biology of the disease and to alter the microenvironment around it, not just to debulk. The evidence supporting maximal safe resection is consistently strong [42,43,44,45,46], but the question driving the next generation of neurosurgical innovation is no longer only how much tumor is removed but also which habitats are visualized, targeted, and rendered accessible to therapy.

Several cross-cutting principles emerge from the translational evidence reviewed here. The first is that intraoperative imaging is most powerful when it reports on tumor biology rather than anatomy alone. 5-ALA does not just help the surgeon take out more tumor. It tells the surgeon which tissue is metabolically active while the operation is still happening [47,48,49,50,51,52,53]. Fluorescein reports on something else entirely: where the blood–brain barrier is breaking down and where vascular permeability is abnormal [54,62,63]. Raman-based platforms ask a different question again: what is this tissue, molecularly, and can we classify it without waiting for the pathology lab [55,56,57,58,64,65]? None of these tools duplicates what the others do. Metabolism, vascular integrity, molecular composition—those are three separate dimensions of tumor biology, and a surgical workflow that captured all three at once would give the surgeon a picture of the microenvironment that no single modality can provide on its own.

Regarding drug delivery, the second principle, which is easy to state but hard to internalize, posits that a drug’s failure in glioma might not stem from the drug itself. The PRECISE trial is the case that makes this argument most convincingly. The agent was biologically rational. The delivery method was CED. The trial was negative [84]. But when Sampson et al. examined what had actually happened with catheter placement and tissue coverage, the answer in many patients was that the drug had simply not reached enough of the target [85].

The therapy did not fail pharmacologically. It failed spatially; the drug never got to enough of the tumor to matter. Nanoparticle-based systems have the same problem in a different form. Preclinical results are strong, but no one has yet shown that these particles reliably cross the BBB in humans or that they end up in the tumor in verifiable concentrations [91,92,93,94,95]. Until delivery technologies are judged by whether they engage their target within the microenvironment, not by systemic drug levels in the blood, this kind of failure will keep happening.

FUS sits in an unusual place among these tools because it may be doing two things at once. The obvious function is opening the barrier so that drugs can get through. But there is accumulating evidence that sonication itself changes the biology of the tissue it touches: immune-cell trafficking, vascular signaling, and local inflammatory tone [75,76,77,79,81]. If that is real, and not just an artifact of preclinical models, then FUS is not a passive delivery aid. It is actively rewriting the microenvironment that the drug enters. The BT008NA trial, which was the first to show a comparative survival signal for microbubble-enhanced FUS combined with temozolomide, lends some clinical weight to that idea [81]. But whether the immunomodulatory and vascular effects of sonication are large enough to matter therapeutically in a broader population has not been established yet.

Where all of this leads, if the technologies mature, is an operating room that does several things in sequence. The surgeon uses 5-ALA to resect metabolically active core disease. SRH classifies tissue at the margin in real time. Perioperative FUS opens the barrier around the infiltrative edge so that systemic or locally infused agents can actually reach the residual cells hiding there [56,79,81]. That is not how glioma surgery works today, but it is not fantasy either. The individual pieces exist. The problem is integration.

Integration, though, is not just a technical challenge. It raises questions about how the field designs trials, measures success, and decides what counts as evidence that a technology works.

Making that work will require more than the technical refinement of any single tool. These technologies developed independently and do not currently communicate with each other within a surgical workflow. Trial design will need to change, too; measuring the extent of resection is not enough when the question is whether a drug reached the right compartment at the right concentration.

How trials measure success is also a problem. Plasma drug concentration says nothing about what is happening inside the tumor. An agent can be circulating at therapeutic levels in the blood while barely penetrating a hypoxic margin or failing entirely to enter the immunosuppressive bed surrounding the resection cavity.

The relevant question was never how they engage with the patient but how much drug is in each part of the tumor and whether being there changed anything. Those are the questions trials should be built around. And trial design has to reckon with the spatial problem directly; the TME is not the same from one region to the next, and any study that treats it as a single pharmacologic target is ignoring the biology that makes glioma so hard to treat in the first place. Most of the technologies discussed in this review are not yet standard of care. But the direction they are heading is unmistakable: glioma surgery is becoming an operation where the surgeon removes disease and interrogates and manipulates the biology of whatever is left behind.

## 5. Limitations

Several limitations of this review and of the technologies it examines warrant consideration.

The most obvious limitation is that the evidence behind these technologies is not all at the same stage. 5-ALA has phase III trial data and is part of routine neurosurgical practice in many centers. Focused ultrasound and implantable CED systems have phase I/II results. Nanoparticle-based delivery is still mostly in animal models [75,76,77,78,79,80,81,91,92,93,94,95]. Putting all of these in the same review is useful for seeing where the field is headed, but it should not be mistaken for an argument that they stand on equal footing. They do not.

Comparing across technologies is also harder than it might appear. The imaging trials do not all define gross total resection in glioblastoma. Postoperative imaging protocols differ. Some studies include only IDH-wildtype glioblastoma, while others are broader. Adjuvant regimens vary. On the delivery side, target populations, dosing, and the methods used to verify that the drug actually reached the tumor are all inconsistent from one trial to the next. Isolating what any one technology contributed to a given outcome is, in most cases, not possible with the data that currently exists. This is an argument for standardized endpoints in future trials, and specifically for endpoints that measure biology and spatial drug distribution, not just imaging volumes.

There are also practical obstacles that have nothing to do with biology. Implantable ultrasound arrays, intraoperative high-field MRI, and chronic CED hardware—these require capital investment, institutional infrastructure, and surgical training that many centers do not have. If any of these technologies proves its worth in large trials, the question of who gets access to it will follow immediately.

The deepest limitation, though, is biological. The glioma microenvironment does not depend on any single signaling axis. It is held together by redundant networks, by phenotypic plasticity in both tumor and immune cells, and by continuous crosstalk between malignant, immune, vascular, and stromal compartments [5,6,7,8,36,37,38,39,40,41]. A technology that disrupts one of those axes—metabolic visualization, barrier opening, localized delivery—may find that the rest of the system compensates. That is the strongest argument for multimodal approaches, and it is also a reason for honesty about where things stand. Comprehensive control of the glioma microenvironment is not something the field is close to achieving.

## 6. Emerging TME Tunable Technologies and Future Directions

The optical and delivery technologies reviewed above are the furthest along clinically, but they are not the whole picture. Several other technology domains are developing in parallel, and any of them could change how glioma surgery is practiced within the next decade.

Artificial intelligence applied to imaging may make it possible to identify tumor habitats, not just tumor boundaries, before and during the operation. Spatial transcriptomics is rewriting the molecular understanding of the TME at a resolution that did not exist five years ago, and that knowledge will eventually filter back into how surgeons plan and execute resections. Sonodynamic therapy borrows the same ultrasound hardware already being tested for BBB disruption, but uses it to treat deep-seated tumor tissue through a different mechanism entirely. Immunotherapeutic delivery systems are being designed to reprogram the immunosuppressive glioma microenvironment locally rather than relying on systemic immune activation. None of these is as clinically proven as 5-ALA or focused ultrasound. But they sit at a point where molecular biology, computational methods, and surgical technology are converging, and what comes out of that convergence could substantially expand what the surgeon is able to do.

### 6.1. Artificial Intelligence-Guided Intraoperative Imaging

The imaging data that surgeons already collect, multiparametric MRI, primarily, contain far more biologic information than is currently being used. Machine-learning algorithms can be trained to find patterns in those sequences that correspond to tumor cellularity, angiogenesis, necrosis, and molecular subtype [50,51,52,53]. The result, in principle, is a way to turn a standard radiologic scan into something closer to a spatial map of tumor biology.

The translational appeal is substantial. AI-based models have demonstrated the ability to predict survival outcomes, infer molecular features, such as IDH mutation status, and delineate imaging habitats associated with aggressive tumor behavior, all from preoperative data already routinely acquired [50,51,52,53]. For the glioma TME, the most consequential application may be the prospective identification of spatially distinct tumor compartments, perivascular, hypoxic, and immunosuppressive, before the surgeon enters the operative field. Integration of radiomic habitat maps with neuronavigation systems could, in principle, allow resection and biopsy strategies to be customized to the biologic architecture of each tumor rather than to its contrast-enhancing boundaries alone.

Current AI models are predominantly trained on retrospective imaging datasets, and almost none of them have been validated prospectively in the context of actual surgical decisions. The features that radiomics extracts are defined differently by different groups. A model built on imaging from one scanner at one institution may not work when applied to data from another. Regulatory frameworks for deploying AI tools intraoperatively barely exist. All of that can be fixed, and it is worth fixing. The molecular biology side of neuro-oncology—spatial transcriptomics and single-cell profiling—is generating extraordinarily detailed maps of tumor biology. Surgeons, meanwhile, operate almost entirely on anatomic information. If AI can close that gap and put molecular context into the surgeon’s hands during the case, it would matter more than most incremental imaging improvements.

### 6.2. Spatial Transcriptomics and Single-Cell Profiling

Bulk RNA sequencing told the field a great deal about which genes are active in glioblastoma, but it homogenized everything. A whole tumor went into the blender, and what came out was an average. Spatial transcriptomics changed that by preserving location. Gene expression could now be mapped to specific coordinates within the tissue, and when that was done, a pattern emerged that no one could see in bulk data. What emerged was geography. The signaling environment next to a blood vessel in a glioblastoma is angiogenic and immunosuppressive next to a necrotic focus; the first is inflammatory and angiogenic, and the second is immunosuppressive and hypoxia-driven. Move to the invasive margin, and the biology shifts again: the stem-like cell populations there have a different character from anything in the tumor core [37,38,39,40,41]. Multi-omic work integrating transcriptomic, proteomic, and metabolomic data has confirmed that these are not artifacts of any single measurement platform. For surgeons, the implication is practical, not abstract.

If different zones of the tumor run on different biology and fail therapy for different reasons, then the surgeon needs tools that can distinguish one zone from another during the operation. This is already done by 5-core in a limited way: bright fluorescence marks the metabolically active core and dimmer fluorescence marks the infiltrative periphery. Raman-based platforms are pushing closer to something that might eventually approximate transcriptomic-level tissue classification during the operation itself [49,53,56,57,64]. The challenge ahead is to close the resolution gap, translating the niche-level molecular detail revealed by spatial transcriptomics into intraoperative signals that surgeons can act on in real time.

Getting there will almost certainly require computational tools that can layer spatial molecular data on top of intraoperative imaging in real time. But if that integration works, the endpoint is something the field has talked about for years without being able to do: tailoring resection boundaries, biopsy targets, and perioperative drug delivery to the molecular identity of the tissue in front of the surgeon, not just to what it looks like on a scan. These approaches may ultimately be integrated into surgical workflows to enable more precise delineation of infiltrative tumor margins and identification of biologically aggressive subregions that are not detectable using conventional imaging alone.

### 6.3. Sonodynamic Therapy

Sonodynamic therapy (SDT) takes a familiar piece of hardware, focused ultrasound, and pairs it with a sonosensitizing compound to generate reactive oxygen species inside tumor tissue [58,59,60,61]. The oxidative damage that results can kill glioma cells and may also injure tumor vasculature. The advantage over photodynamic therapy is physical: light does not penetrate deep brain tissue well, but ultrasound does, which makes SDT applicable to tumors that phototherapy simply cannot reach.

What makes SDT interesting from a microenvironment standpoint is that it may not be purely cytotoxic. SDT can damage the angiogenic architecture of tumor vasculature and the immune-cell composition within treated tissue [58,59,60,61]. That combination is what makes SDT worth watching. A therapy that kills glioma cells is useful. A therapy that also damages the tumor’s blood supply and reshuffles the immune landscape of the treated zone is a different proposition—it is not just cytotoxic, it is microenvironmentally disruptive. The mechanism is nothing like FUS-mediated BBB opening, but the conceptual territory overlaps: both use ultrasound to change the biology of the tissue, not just to deliver or destroy.

Clinical data so far are limited to early-phase trials combining SDT with temozolomide in recurrent glioblastoma. Safety has been acceptable. Efficacy, durability, optimal sonosensitizer choice, dosimetry, and treatment scheduling are all still open. And the bar SDT has to clear is not static; it rises every time FUS-mediated BBB disruption posts another positive result.

### 6.4. Immunotherapeutic Delivery Platforms

Immunotherapy has transformed outcomes in several solid tumor types. Glioblastoma is not one of them. The microenvironment is the main reason. TAMs flood the tumor with immunosuppressive cytokines. Innate checkpoint pathways—CD47–SIRPα, CD24–Siglec-10—block phagocytic clearance. T-cells that do infiltrate the tumor become exhausted. And the BBB limits how many immune cells get in to begin with [15,16,17,18,19,20,21,22]. Systemic immunotherapy runs into all of these barriers at once, which is why the results in glioblastoma have been so disappointing relative to other cancers. The logic of local delivery follows directly from that failure: if the immune microenvironment cannot be reprogrammed from outside the brain, the reprogramming agents need to be delivered to the tumor site itself (Table 3).

The platforms being tested for this are varied. Nanoparticles can be loaded with cytokines, checkpoint inhibitors, or immunostimulatory adjuvants and directed into the tumor bed or into TAM-dense regions of the microenvironment [91,92,93,94,95]. Oncolytic viruses offer a different approach: they replicate preferentially in tumor cells, lyse them, and, in doing so, scatter immunogenic debris that can recruit and activate immune effector populations.

### 6.5. Future Directions

The technologies in this review sit at different distances from routine clinical use, but they face a set of common problems that will determine whether any of them gets there.

The first is interoperability. 5-ALA, Raman-based imaging, focused ultrasound, and CED were each developed in isolation. No existing surgical platform integrates metabolic fluorescence, real-time tissue classification, and perioperative barrier disruption into a single workflow. Building that integration, deciding what information feeds into what decision, and in what order, is an engineering problem the field has not yet seriously taken on.

The second is trial design. Extent of resection has been the dominant neurosurgical endpoint for decades, and it has served the field well. But it cannot capture whether a drug reached the right compartment, whether the local microenvironment was altered, or whether a biologic signal changed the surgeon’s plan in a way that mattered for the patient. Future trials will need spatial and biologic endpoints alongside volumetric ones, and the statistical frameworks for evaluating those endpoints largely do not exist yet.

The third is access. Implantable ultrasound devices, intraoperative high-field MRI, chronic CED hardware, and SRH platforms require capital, infrastructure, and training that are concentrated in a small number of academic centers. If these technologies prove effective, the question of how to make them available broadly will become unavoidable.

None of these problems is a reason to slow down. All of them are reasons to be deliberate about what comes next.

## 7. Conclusions

The glioma microenvironment is not an abstraction. It is the vasculature that controls where drugs go, the immune landscape that determines whether the tumor is surveilled or protected, and the barrier architecture that dictates which cells are exposed to therapy and which are not. Every technology reviewed here, from 5-ALA to focused ultrasound to the early-stage platforms still working through preclinical validation, engages one or more of those features directly.

What has changed is ambition. Glioma surgery used to ask a single question: how much tumor can be safely removed? That question still matters. But 5-ALA proved that a metabolic signal, made visible during the operation, could change what the surgeon removed and improve how long patients survived. Focused ultrasound is showing that changing where drugs go inside the brain changes what those drugs can do. The principle is no longer hypothetical.

The question now is scope. How many dimensions of the microenvironment can the surgeon eventually read, reach, and alter in the course of a single operation? The answer to that question will define the next generation of glioma surgery.

## Figures and Tables

**Table 1:** Summary of major clinical studies evaluating fluorescence-guided surgery in malignant glioma. GTR—gross total resection; PPV—positive predictive value; HGG—high-grade glioma; 5-ALA—5-aminolevulinic acid.

Study	Population	Type of Study	Intervention	Control	Key Results (Intervention vs. Control)
Stummer Phase III study (2006) [48]	322 patients with malignant glioma	Randomized controlled trial	20 mg/Kg bodyweight 5-ALA (n = 161)	Conventional microsurgery with white light (n = 161)	- Higher complete resection of contrast-enhancing tumor by 29% - Higher 6-month progression-free survival (41.0% vs. 21.1%) - No difference in adverse effects within 7 days post-op
Phase II FLUOGLIO study (2014) [62]	20 patients	Phase II randomized controlled trial	5–10 mg/kg fluorescein IV (n = 20)	NA	- Complete resection of contrast-enhanced tumor in 80% of patients - 6-month progression-free survival rate of 71.4%
Neira et al. (2016) [63]	32 patients	Prospective observational translational study	3 mg/kg fluorescein sodium IV (n = 32)	Internal tissue controls (different tumor regions)	- GTR in 84% of patients with resection average of 95%
Schupper et al. (2021) [71]	69 patients providing 275 tumor samples	Single-arm, multicenter prospective study	5-ALA (n = 71)	NA	- Sensitivity of 9.65%; specificity of 29.4% - PPV of HGG histopathology of 95.4% and diagnostic accuracy of 92.4% - Drug-related adverse outcomes occurred in 22%
Katsevman et al. (2019) [72]	57 patients	Retrospective chart review	Sodium fluorescein (n = 57)	NA	- Complete or near-total (>98%) resection achieved in 73%

**Table 2:** Comparison of emerging strategies to overcome the blood–brain barrier (BBB) for drug delivery in brain tumors. BBB—blood–brain barrier; TME—tumor microenvironment; EPR—enhanced permeability and retention.

	Focused Ultrasound-Mediated BBB Disruption	Convection-Enhanced Delivery	Nanoparticle-Based Drug Delivery
Maneuvering the BBB	Opens the BBB locally and reversibly.	Goes around the BBB.	BBB remains closed.
Mechanism behind technique	Oscillation of microbubbles caused by ultrasound widens tight junctions, allowing entry to therapeutic agents.	Use of catheter to deliver therapeutic agents directly in brain parenchyma using positive pressure.	Nanoparticles designed to cross the BBB can be tuned to mimic biological conditions of TME and accumulate in tumor tissue.
Advantages compared to systemic chemotherapy	Increases concentrations of therapeutic agents within the intratumoral region.	Ability to control placement of therapeutic agent into tissue with residual disease.	Development of nanoparticles to locate niche areas of immune suppression as a target.
Concerns regarding delivery technique	Variability of penetration of therapeutic agents across BBB. Optimal sonication parameters, extent and uniformity of BBB opening still require more research.	Therapeutic agents may appear ineffective if relevant tumor habitat is not breached.	EPR effect of therapeutic agents is less reliable as BBB integrity varies across tumor landscapes. Off-target accumulation of nanoparticles. Clearance of nanoparticles by reticuloendothelial system.

**Table 3:** Studies on immunotherapeutic targets and delivery platforms in glioblastoma. BBB—blood–brain barrier; TME—tumor microenvironment; TAMs—tumor-associated macrophages; CSF-1R—colony-stimulating factor 1 receptor; mTOR—mechanistic target of rapamycin; PLGA—poly(lactic-co-glycolic acid); EPR—enhanced permeability and retention; GBM—glioblastoma multiforme.

Study	Model/System	Immunologic Target	Delivery Strategy	Key Finding	Relevance to GBM Therapy
Pyonteck et al. (2013) [15]	Murine glioma models	CSF-1R signaling in TAMs	CSF-1R inhibition	Reprogrammed macrophages from tumor-supportive to tumor-inhibitory phenotype	Established macrophage polarization as a therapeutic target in GBM
Dumas et al. (2020) [17]	Mouse and human GBM samples	Microglial mTOR signaling	Pharmacologic and genetic inhibition	Microglia drove immunosuppression through mTOR signaling	Highlighted microglial signaling as a therapeutic target
Hutter et al. (2019) [20]	Mouse GBM models	CD47–SIRPα innate checkpoint	Anti-CD47 antibody	Microglia mediated tumor phagocytosis after checkpoint blockade	Demonstrated innate immune checkpoint targeting in brain tumors
Barkal et al. (2019) [21]	Human tumor models	CD24–Siglec-10 checkpoint	Antibody blockade	Blocking CD24 restored macrophage phagocytic activity	Identified additional innate checkpoint pathway relevant to GBM
Blanco et al. (2015) [91]	Nanomedicine framework	Biological barrier penetration	Nanoparticle design principles	Particle size and charge determined delivery success	Foundational guidance for BBB-penetrating drug systems
Zhao et al. (2024) [94]	Preclinical GBM nanomedicine studies	Tumor targeting and immune microenvironment	Lipid-based nanoparticles	Improved BBB penetration and targeting of GBM stem cells	Supports nanoparticle-mediated local immunotherapy

## Data Availability

No new data were created or analyzed in this study.
